# A Comprehensive Investigation of Active Learning Strategies for Conducting Anti-Cancer Drug Screening

**DOI:** 10.3390/cancers16030530

**Published:** 2024-01-26

**Authors:** Priyanka Vasanthakumari, Yitan Zhu, Thomas Brettin, Alexander Partin, Maulik Shukla, Fangfang Xia, Oleksandr Narykov, Michael Ryan Weil, Rick L. Stevens

**Affiliations:** 1 Division of Data Science and Learning, Argonne National Laboratory, Lemont, IL 60439, USA; yitan.zhu@anl.gov (Y.Z.); apartin@anl.gov (A.P.); mshukla@anl.gov (M.S.); fangfang@anl.gov (F.X.); onarykov@anl.gov (O.N.); 2 Computing, Environment and Life Sciences, Argonne National Laboratory, Lemont, IL 60439, USA; brettin@anl.gov (T.B.); stevens@anl.gov (R.L.S.); 3 Cancer Research Technology Program, Cancer Data Science Initiatives, Frederick National Laboratory for Cancer Research, Frederick, MD 21701, USA; ryan.weil@nih.gov; 4 Department of Computer Science, The University of Chicago, Chicago, IL 60637, USA

**Keywords:** active learning, machine learning, drug response prediction, drug discovery, cancer

## Abstract

**Simple Summary:**

Preclinical drug screening experiments for anti-cancer drug discovery typically involve testing candidate drugs against cancer cell lines. This process can be expensive and time consuming since the possible experimental space can be quite huge, involving all of the combinations of candidate cell lines and drugs. Guiding drug screening experiments with active learning strategies could potentially identify promising candidates for successful experimentation. This study investigates various active learning strategies for selecting experiments to generate response data for identifying effective treatments and improving the performance of drug response prediction models. We have demonstrated that most active learning strategies are more efficient than random selection for identifying effective treatments.

**Abstract:**

It is well-known that cancers of the same histology type can respond differently to a treatment. Thus, computational drug response prediction is of paramount importance for both preclinical drug screening studies and clinical treatment design. To build drug response prediction models, treatment response data need to be generated through screening experiments and used as input to train the prediction models. In this study, we investigate various active learning strategies of selecting experiments to generate response data for the purposes of (1) improving the performance of drug response prediction models built on the data and (2) identifying effective treatments. Here, we focus on constructing drug-specific response prediction models for cancer cell lines. Various approaches have been designed and applied to select cell lines for screening, including a random, greedy, uncertainty, diversity, combination of greedy and uncertainty, sampling-based hybrid, and iteration-based hybrid approach. All of these approaches are evaluated and compared using two criteria: (1) the number of identified hits that are selected experiments validated to be responsive, and (2) the performance of the response prediction model trained on the data of selected experiments. The analysis was conducted for 57 drugs and the results show a significant improvement on identifying hits using active learning approaches compared with the random and greedy sampling method. Active learning approaches also show an improvement on response prediction performance for some of the drugs and analysis runs compared with the greedy sampling method.

## 1. Introduction

In the year 2023, 1.96 million new cases of cancer are projected to be reported, with more than half a million deaths [1]. Cancer is a highly heterogenous disease and two patients with cancer affecting the same physiological location may require different specialized treatments to control the tumor progression [2,3]. Thus, drug response prediction becomes an important task, the success of which can assist precision medicine, which allows healthcare providers to offer personalized treatment after a comprehensive genomic analysis of the patient’s cancer cells [4,5]. Drug response prediction models [6] are designed to predict the effectiveness of a particular drug in treating a patient’s cancer. The models are trained on cancer representations and/or drug representations to predict the response of the cancer to the drug under consideration. The cancer representations can be genomic signatures such as gene expressions, copy number variations, mutations, and DNA methylations, or pathology images. Drug representations can be molecular fingerprints, drug descriptors, SMILES strings, or graphical representations. The response to drug treatment can be measured by the half maximum inhibitory concentration (IC50), the area under the dose response curve (AUC), the area above the dose response curve (AAC), etc.

Over the past decade, many anti-cancer drug response prediction models have emerged using both traditional machine learning algorithms as well as more sophisticated deep learning techniques [6,7]. Examples of conventional machine learning algorithms employed for anti-cancer drug response prediction include linear regression [8], support vector machine (SVM) [9,10], random forests (RF) [3,11,12], and boosting-based methods [13,14]. MOLI [15], DrugOrchestra [16], PathDSP [17], and several other models [18] use fully connected neural networks to predict drug responses of cancer cell lines represented by their genomic signatures. GraphDRP [19], tCNNs [20], and DeepCDR [21] are representative drug response prediction models utilizing convolutional neural networks (CNN) in their model architectures. Paccmann [22], DeepTTA [23], HiDRA [24], GraTransDRP [25], and CADRE [26] employ attention-based neural networks in their model architectures, which help identify important genes through the self-attention mechanism. 

Cancer drugs undergo very intense and elaborate drug screening protocols before they can be approved for clinical use [27,28]. The US Food and Drug Association (FDA) approved 332 new anti-cancer drugs between the years 2009 and 2020 [28]. Pre-clinical drug screening typically involves testing drugs against known cancer cell lines followed by animal model testing. There are more than 1000 cancer cell lines considered in the Cancer Cell-Line Encyclopedia (CCLE) project [29]. The experimental space for preclinical drug screening against cell lines can be quite huge. For example, choosing experiments for drug repurposing could mean testing the 332 drugs approved by the FDA against all available cancer cell lines. Performing experiments to exhaustively search all or a significant portion of possible combinations can be prohibitively expensive and time consuming. A potential solution to this challenge is drug screening experiments guided by response modeling via active learning [30,31,32,33,34,35]. Drug response prediction with active learning tries to efficiently build high-performance response prediction models with limited drug screening data while simultaneously discovering a large amount of validated responsive treatments. 

Active learning is an iterative machine learning procedure, in which the model learning process is divided into iterations and in each iteration a group of new samples is selected based on a designed strategy and added to the model training dataset [32,36,37]. In each iteration of the active learning process, the current model is used to generate predictions on all unlabeled data points. These predictions can be utilized to select samples from the unlabeled set to generate annotations/ground truth labels, which in drug screening experiments are the treatment response measurements. These newly annotated samples are then added to the training data to build the model in the next iteration. In comparison to annotating randomly selected samples for model training, active learning can usually achieve a superior model performance with fewer training samples, thus saving considerable data annotation cost [38,39,40,41].

Active learning has been used in many computer vision applications [37] such as autonomous navigation [42,43], and biomedical image analysis [40,44]. Autonomous navigation systems require enormous amount of data as images or point clouds to ensure reliable and safe operations. Active learning helps to save considerable data collection and annotation costs by intelligently choosing training data. Medical images such as histopathology images require expert knowledge to generate annotation, which is also tedious and time-consuming work. Active learning saves a considerable amount of work by iteratively recommending samples to be annotated, so that a well-performing model can be generated with a relatively limited amount of annotated data [41]. Table 1 summarizes some of the published works using active learning in several application domains.

Active learning is a very useful technique especially in biomedical applications [31,34,40], where the cost of experimentation to collect data labels is high. It has been used in drug discovery applications to identify suitable drug candidates. For drug screening experiments, active learning can help to identify effective treatments much earlier in the process, thereby saving substantial time and resources [31,32,33,34,35]. Previous studies have demonstrated the use of active learning strategies in selecting experiments for protein-drug activity measurement by quantitative structure activity relationship (QSAR) analyses [32,33]. However, there are very limited existing works of using active learning strategies for anti-cancer drug screening. To the best of our knowledge, there has been only one work investigating active learning for anti-cancer drug response prediction [30]. However, this work evaluates the capability of the technique in identifying responsive treatments, while the model performance on response prediction has not been thoroughly studied and compared with baselines.

**Table 1:** Summary of published works using active learning in several application domains.

Reference	Approach	Application
[41]	Monitors the normalized average loss and normalized average predictive entropy of every sample. Eliminates noisy samples and selects the most informative samples for annotation.	Histopathology image analysis
[44]	Queries unlabeled samples that maximize the average distance to training set samples.	Medical image analysis
[45]	Uncertainty sampling identifies the next set of sentences to be annotated.	Natural language processing
[42]	Diversity-based active learning to annotate the most informative frames and objects.	Autonomous navigation and object detection
[46]	Utilizes Bayesian global optimization (BGO) to select an experiment by maximizing a utility function.	Material science
[47]	Selects samples from the unlabeled set using uncertainty computed by a discrete information entropy measure.	Industrial fault detection
[48]	Uses diversity-based sampling and loss-prediction sampling to select unlabeled lung CT image samples for annotation.	Disease diagnosis (COVID-19)
[49]	Reduces annotations at both image-level and pixel-level using uncertainty-based active learning. Uncertainty is estimated by computing entropy at the image and pixel levels.	Semantic segmentation
[33]	Used uncertainty, greedy, and random active learning workflows for predicting drug responses.	Drug response prediction

In this work, active learning strategies are implemented and investigated for drug-specific anti-cancer response prediction, in which a prediction model is constructed for each drug to predict its treatment effect on various cancer cell lines. Several sampling techniques such as random, greedy, uncertainty-based, and diversity-based methods, and their hybrid approaches have been investigated. This work summarizes the results of applying all different sampling techniques separately for 57 drugs over cancer cell lines. The number of cancer cell lines tested for the drugs varied from 501 to 764. The techniques have been evaluated and compared based on two measures: the early identification of responsive treatments (i.e., hits) and early improvement on model prediction performance. Making early progress on these two goals enables the active learning process to stop sooner, achieving comparable results with reduced reliance on obtaining labeled data. 

Our study has made several unique contributions to the research field. First, it is a pioneering work performing a comprehensive investigation on multiple active learning techniques for anti-cancer drug response prediction. The only existing work of applying active learning to anti-cancer drug response built cell line-specific models to predict the response of a specific cell line to various drug-pair treatments [30]. Differently, our study builds drug-specific models to predict the responses of various cell lines to a specific single-drug treatment. Our study investigates the performance of active learning strategies for both hit detection and drug response modeling, while the previous work mainly focused on hit detection [30]. Second, we have designed and implemented multiple active learning strategies using different sampling techniques for a comprehensive evaluation and comparison. Third, we have devised a set of novel experimental procedures and performance metrics to evaluate active learning approaches for anti-cancer drug response modeling. Fourth, through our analysis, we have demonstrated that active learning can substantially enhance the identification of responsive treatments. Additionally, we have observed its beneficial impact on response modeling for certain experimental settings compared to pure greedy approaches.

## 2. Materials and Methods

### 2.1. Data Sources and Data Splitting

We conducted the active learning analysis on a large cell line drug screening dataset, the Cancer Therapeutics Response Portal v2 (CTRP) [50], which includes 494 drugs, 812 cell lines, and 318,040 experiments. Here, experiments refer to the unique combinations of drugs and cell lines. For each experiment, we fitted a dose–response curve to the multi-dose viability measurements and calculated a normalized area under the dose response curve (AUC_res) for the dose range of [10^−10^ M, 10^−4^ M] as the response measure. For drug response modeling, cell lines were represented by gene expression profiles generated using RNA-seq. TPM (transcripts per million reads mapped) values were calculated as expression values (x), which are log2(x + 1) transformed and then standardized so that each gene has a zero mean and a unit standard deviation across cell lines. For the analysis, we used only the 943 “landmark” genes identified in the Library of Integrated Network-Based Cellular Signatures (LINCS) project, which have been shown to provide a good representation of cellular transcriptomic changes [51]. For each drug, we built a model to predict the responses of various cell lines under the treatment of this drug, the input data of response models are cell line gene expression profiles, and the output is the predicted AUC_res values indicating the responses of cell lines to the drug treatment. 

A subset of 57 drugs was chosen for the study based on three criteria. Table S1 in the Supplementary Materials contains a list of all of the drugs used along with their Mechanisms of Action (MoA). Firstly, a drug needs to be tested in experiments against at least 500 cancer cell lines, to guarantee a good number of experiment samples for building a response model. Secondly, a drug needs to provide effective treatments (AUC_res < 0.5) for at least 20 cell lines to guarantee the existence of sufficient hits for response modeling. Thirdly, the proportion of hits in experiments must not exceed 70% to exclude highly toxic compounds. The number of cell lines treated by a drug in the selected subset varies from 501 to 764. We have built and evaluated drug-specific response prediction models through active learning separately for each drug within the selected subset. 

**Figure 1:**
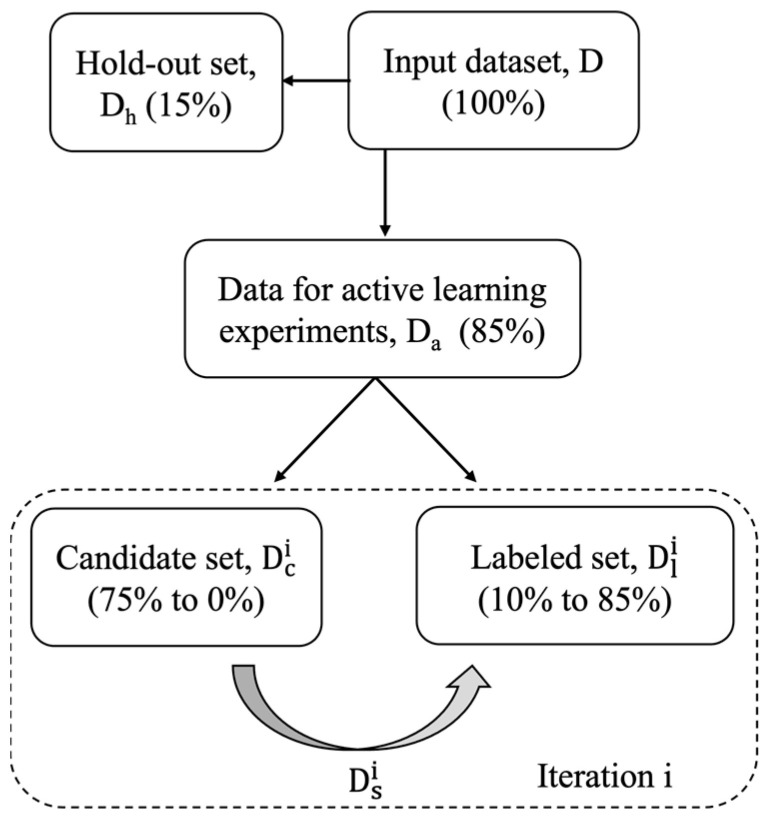
Schematic of data splitting for active learning analysis.

**Figure 2:**
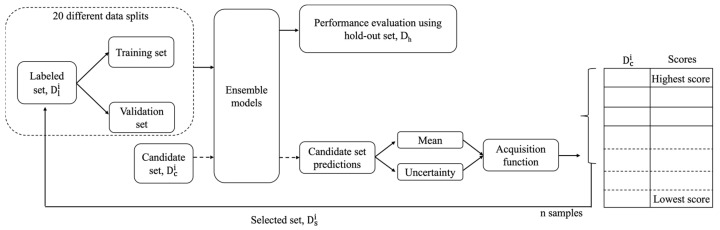
Workflow of active learning analysis for iteration i. Diversity-based and random sampling approaches do not make predictions on the candidate set and follow a similar but simplified workflow.

Figure 1 shows the data splitting strategy for conducting the active learning analysis of drug response prediction. The input dataset D, for each drug, consists of the gene expression profiles of cell lines against which the drug is tested, and the labels are the AUC_res response values for the pairs of drugs and cell lines. D is split into a dataset for conducting active-learning analysis denoted by D_a_ and a holdout set denoted by D_h_. The holdout set D_h_ is used for testing the model prediction performance at each iteration of the active learning process and contains 15% of samples randomly chosen from D. After determining the holdout set, 10% of samples from D_a_ are randomly selected to initialize the labeled set $D_{l}^{i}$ for model training, while the rest 75% of the samples initializes the candidate set $D_{c}^{i}$, where i is the iteration index starting from 1. The active learning cycle is iteratively executed using $D_{l}^{i}$ and $D_{c}^{i}$ (Figure 2). In each iteration, a subset of $D_{c}^{i}$, denoted as $D_{s}^{i}$, is selected and labeled, and will be combined with $D_{l}^{i}$ in the next iteration to form $D_{l}^{i+1}$. For each drug, the active learning process is repeated 50 times with different splits of D_h_ and D_a_, to ensure a robust result evaluation.

### 2.2. Active Learning Approaches and Workflow

Figure 2 shows the workflow of the active learning analysis. In each iteration i, the labelled set $D_{l}^{i}$ is split twenty times to generate 20 different pairs of training and validation sets, where the validation set is 15% of $D_{l}^{i}$. A total of 20 machine learning models are trained separately on these 20 training sets and the corresponding validation sets are used for hyperparameter optimization and the early stopping of model training. This generates an ensemble of 20 prediction models. The purpose of using an ensemble of models is to estimate the uncertainty of predictions generated by the model. The models are then tested on the candidate set $D_{c}^{i}$ to make predictions. Some active learning approaches select $D_{s}^{i}$ from the candidate set by ranking samples based on the scores computed using an acquisition function that can take the prediction values into consideration. Several acquisition functions are used in this work to calculate the score of each sample using the mean (μ) and/or standard deviation (σ) of prediction values from the ensemble of models. The standard deviation of prediction values serves as a measure of uncertainty. Finally, n samples from $D_{c}^{i}$ with the highest scores are chosen to form the selected set $D_{s}^{i}$, which is added to the labelled set $D_{l}^{i}$ to produce $D_{l}^{i+1}$ for the next iteration. The samples in $D_{s}^{i}$ are also simultaneously removed from $D_{c}^{i}$ to produce $D_{c}^{i+1}$ used in the next iteration. The value of n considered in all of the analyses is 20. This process is repeated until the entire candidate set is exhausted and added to the labelled set. So, the last iteration may have fewer than 20 samples depending on the number of remaining samples available. 

The approaches used for selecting samples in this work are as follows:

1. Greedy [30,33]: This approach uses an acquisition function $F\left(x\right)=-\mu \left(x\right)$, which considers only the mean of the prediction values generated by the 20 different models for a candidate sample. The negative sign allows the acquisition function to give a large value to a candidate sample with low AUC predictions, as a low AUC value indicates a responsive treatment.
2. Uncertainty [47,49,52]: This approach uses an acquisition function $F\left(x\right)=\sigma \left(x\right)$, which considers only the prediction uncertainty.
3. GU combined [30]: This approach uses an acquisition function $F\left(x\right)=-\mu \left(x\right)+\sigma \left(x\right)$, which is a combination of greedy and uncertainty.
4. Diversity [42,48]: This approach does not consider predictions on the candidate set $D_{c}^{i}$. It is based on the diversity of samples in $D_{c}^{i}$. K-means clustering is performed on $D_{c}^{i}$ with the cluster number equal to ‘n’. Then in every cluster, the sample closest to the cluster centroid is chosen and added to the labelled set $D_{l}^{i}$.
5. Random [30,33]: The samples added to the labelled set $D_{l}^{i}$ are chosen randomly from the candidate set $D_{c}^{i}$. This approach is primarily used as a baseline to compare with all the other approaches.

In each iteration, the prediction performance of a trained model is evaluated using the holdout set. For the rest of this paper, all sampling approaches except ‘Random’ will be mentioned as active learning approaches.

Two hybrid approaches are implemented to investigate the effect of combining the GU combined active learning acquisition function with random sampling.

- Hybrid sampling: In every iteration of the analysis, the ‘ps’ percentage of $D_{s}^{i}$ samples is selected using random sampling. The remaining samples are selected using the GU combined acquisition function. The values of ‘ps’ are 20%, 30%, 40%, and 50%.
- Hybrid iteration: In this approach, random sampling is used in the initial ‘pi’ percentage of iterations, while the remaining iterations use the GU combined acquisition function. The values of ‘pi’ are 20%, 30%, 40%, and 50%.

The total number of analyses conducted for each drug is thirteen. We use the sampling approach names as the names of analyses, for example, greedy, random, uncertainty, GU combined, and diversity analyses. The analyses applying hybrid sampling are called hybrid sampling—0.2, hybrid sampling—0.3, hybrid sampling—0.4, and hybrid sampling—0.5 for 20%, 30%, 40%, and 50% of added samples in each iteration being randomly chosen, respectively. The analyses applying the hybrid iteration approach are called hybrid iteration—0.2, hybrid iteration—0.3, hybrid iteration—0.4, and hybrid iteration—0.5 for the initial 20%, 30%, 40%, and 50% of iterations using random sampling, respectively. 

### 2.3. Prediction Model

The machine learning model considered in this work is LightGBM, a decision tree-based gradient boosting algorithm. LightGBM algorithm has been implemented using the LightGBM python package, version 3.2.1. The maximum number of leaves in each decision tree is 31 with a learning rate of 0.05 and mean square error as the loss function. The maximum number of boosting rounds is 500 for model training, and early stopping happens if the loss on validation set does not reduce in 30 consecutive rounds.

### 2.4. The Definitions and Demonstrations of Active Learning Performances

**Figure 3:**
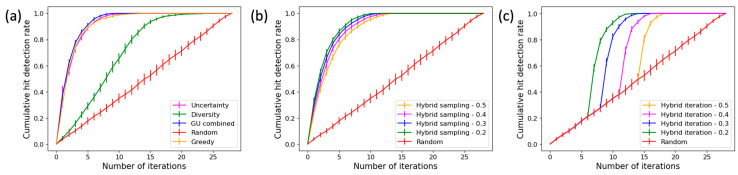
Curves of cumulative hit detection rate for different sampling methods. (**a**) Multiple active learning methods, (**b**) hybrid sampling methods, and (**c**) hybrid iteration methods. Random sampling is included in all three plots as a baseline for comparison purposes.

The performance of an active learning approach is evaluated from two aspects: (1) the rate of detecting experimentally validated hits and (2) the rate of improving drug response prediction performance. An experimentally validated hit is a cell line in the selected set $D_{s}^{i}$ with an AUC value < 0.5. If one active learning approach can detect a higher number of hits early on compared with another approach, it has a superior performance in terms of detecting hits, because with the same number of experiments it can identify more experimentally validated hits. The performance of detecting hits early on can be quantified by the normalized area under the curve of the cumulative hit detection rate, which is denoted by AUC_hit_. The cumulative hit detection rate is defined as

$$r_{i}=\frac{\sum_{j=1}^{i}h\left(D_{s}^{j}\right)}{h\left(D_{c}^{0}\right)}$$

where $r_{i}$ is the cumulative hit detection rate at iteration *i*, $h\left(\cdot \right)$ is a function returning the number of hits in a sample set, and $D_{c}^{0}$ is the initial candidate set at the beginning of analysis. We then calculate AUC_hit_ by

$${AUC}_{hit}=\frac{\sum_{i=1}^{I}r_{i}}{I}$$

where *I* is the total number of iterations. The value of AUC_hit_ is in the range of [0, 1]. We take the drug cytarabine as an example and show in Figure 3 the curves of cumulative hit detection rate for different approaches. A high AUC_hit_ value indicates a sampling method can help identify hits early on. Since the analysis of each sampling method is conducted 50 times with different data partitions, the average cumulative hit detection rate and associated standard deviation are measured at each iteration and shown in Figure 3.

**Figure 4:**
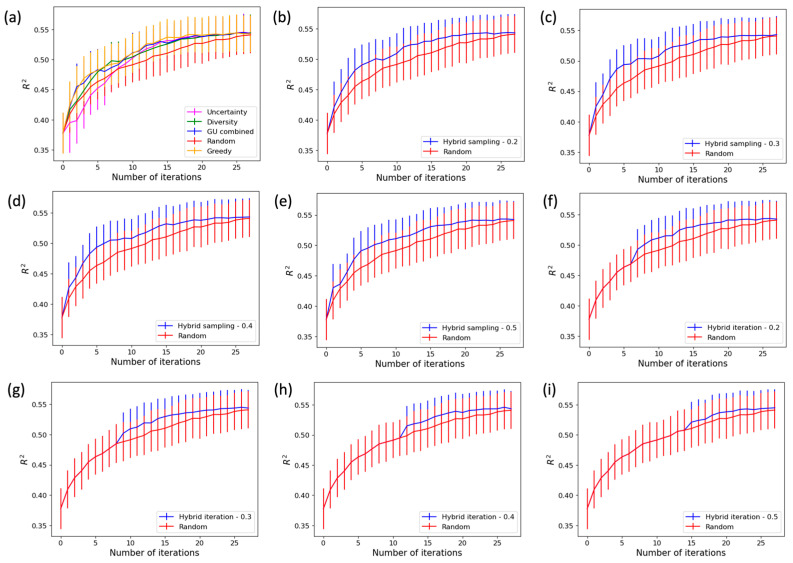
Model performance comparison between different sampling methods. (**a**) Multiple active learning methods, (**b**) hybrid sampling with ps = 20%, (**c**) hybrid sampling with ps = 30%, (**d**) hybrid sampling with ps = 40%, (**e**) hybrid sampling with ps = 50%, (**f**) hybrid iteration with pi = 20%, (**g**) hybrid iteration with pi = 30%, (**h**) hybrid iteration with pi = 40%, and (**i**) hybrid iteration with pi = 50%. Performance of random sampling is shown in every plot for comparison.

In each iteration of the analysis process, the drug response prediction performance of a model is evaluated on the holdout set D_h_, which is quantified by the R-squared (R^2^) value. Figure 4 shows the curves of model prediction performance across iterations for different sampling methods. The normalized area under the R^2^ curve, denoted by AUC_per_, can be used for quantifying how quickly the model prediction performance improves during the active learning process. The AUC_per_ is calculated as

$${AUC}_{per}=\frac{\sum_{i=1}^{I}p_{i}}{I}$$

where $p_{i}$ is the R^2^ prediction performance of the model at iteration *i*. The faster a particular approach improves the model prediction performance, the higher the AUC_per_ value will be. 

## 3. Results

### 3.1. Comprehensive Hits Analysis

**Figure 5:**
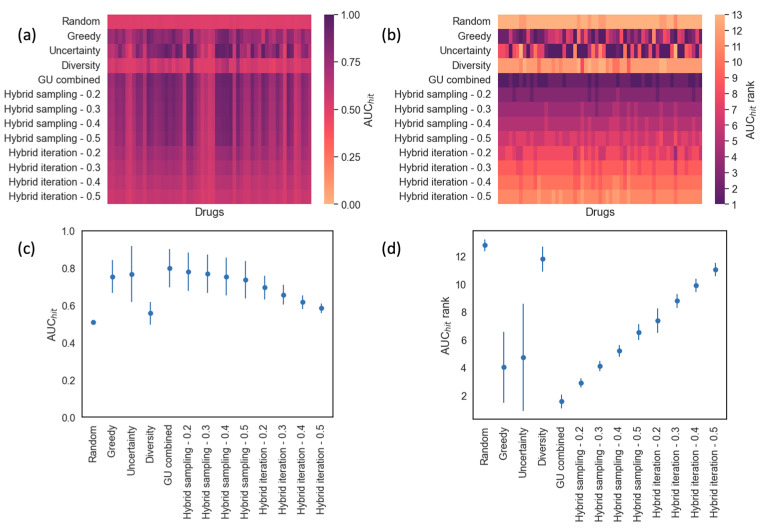
Hit analysis over 57 drugs. (**a**) Heat map showing the average AUC_hit_ obtained for all active learning methods and drugs. (**b**) Heat map showing the AUC_hit_ rank for each drug across all active learning methods. Methods with high AUC_hit_ values for a drug receive low AUC_hit_ ranks. (**c**) Scatter plot showing the mean and standard deviation of AUC_hit_ over all of the drugs for each method. (**d**) Scatter plot showing the mean and standard deviation of AUC_hit_ ranks obtained for each method across all drugs.

The area under the cumulative hits curve (AUC_hit_) is used as a metric in determining which sampling approach is better than the other in detecting hits. The results of the analyses conducted with all 57 drugs are summarized using heatmaps and scatter plots, shown in Figure 5. Figure 5a shows the average AUC_hit_ for each sampling method and drug. The more purple areas indicate higher AUC_hit_ values. In order to determine which sampling methods identify hits faster, the methods are ranked based on the AUC_hit_ values for each drug to generate AUC_hit_ ranks. The method with the highest AUC_hit_ value is ranked 1 and the analysis with the lowest AUC_hit_ value is ranked 13. Figure 5b shows the heatmap indicating the AUC_hit_ ranks assigned for each method over all drugs. The more purple areas indicate methods and drugs with lower AUC_hit_ ranks, hence higher AUC_hit_ values. Figure 5c,d show the means and standard deviations across all drugs for the AUC_hit_ values and ranks, respectively. These plots help to understand if a particular sampling method is generally better than the other in identifying hits across all drugs in consideration. 

As shown in Figure 5, ‘Diversity’ and ‘Random’ analyses are not very efficient in identifying hits. However, ‘GU combined’, ‘Greedy’, ‘Uncertainty’, and ‘Hybrid sampling’ analyses can identify hits more quickly. This trend is more evident from the scatter plots in Figure 5d, where the mean ranks for ‘Random’ and ‘Diversity’ are around 12, which means that these analyses were mostly ranked last in terms of identifying hits over all of the drugs. ‘GU combined’ performs the best among all competing methods, including ‘Greedy’ and ‘Uncertainty’. This is particularly interesting as the acquisition function of ‘GU combined’ is basically a combination of those of ‘Greedy’ and ‘Uncertainty’, which helps to identify more hits early on in comparison with using the acquisition functions individually.

To examine whether a method performs better than random or greedy sampling, two-tail pair-wise *t*-tests were conducted to compare every method with either ‘Greedy’ or ‘Random’. The obtained results are shown in Table 2. A *p*-value < 0.05 implies that the two methods produce significantly different results in hit detection. A positive mean AUC_hit_ difference indicates that the method in consideration (indicated in the first column of Table 2) performs better than the baseline method (either ‘Greedy’ or ‘Random’); otherwise, the method in consideration performs worse. All of the entries in Table 2 with *p*-value < 0.05 and positive mean AUC_hit_ differences are indicated in bold.

Furthermore, pairwise Wilcoxon signed-rank tests were conducted based on AUC_hit_ ranks between each method and a baseline approach (either ‘Greedy’ or ‘Random’). The *p*-values and the differences in the mean ranks are shown in Table 3. A positive mean rank difference indicates the method in consideration performs better than either ‘Random’ or ‘Greedy’. Similar to Table 2, all of the methods with *p*-values < 0.05 and positive mean AUC_hit_ rank differences are indicated in bold.

‘GU combined’ is the only method outperforming ‘Greedy’ with statistically significant *p*-values (<0.05) from both t-test and Wilcoxon signed-rank test shown in Table 2 and Table 3. This indicates that the acquisition function combining greedy and uncertainty sampling is more helpful in identifying hits than using the pure ‘Greedy’ acquisition function. ‘Hybrid sampling—0.2’ also showed a better average AUC_hit_ rank when compared to ‘Greedy’, while all other methods showed lower performance in identifying hits in comparison to ‘Greedy’. Comparing between hybrid sampling with different ps values, more utilization of random sampling, indicated by higher ps values, reduces the hit identification performance. The same pattern is observed for hybrid iteration methods with different pi values. Basically, the lower the contribution of random sampling is (i.e., smaller ps and pi values), the higher the difference in AUC_hit_ is when compared with ‘Greedy’. This observation is consistent with the finding that random sampling provides the lowest performance among all methods as demonstrated by all positive values in the last columns in Table 2 and Table 3. Table 2 and Table 3 demonstrate that all methods statistically significantly outperform ‘Random’ with *p*-values < 0.05. This is a very important observation as we can say that all of the active learning approaches can identify higher numbers of hits much earlier in the process than randomly selecting experiments. 

### 3.2. Comprehensive Analysis on Drug Response Modeling Performance 

**Figure 6:**
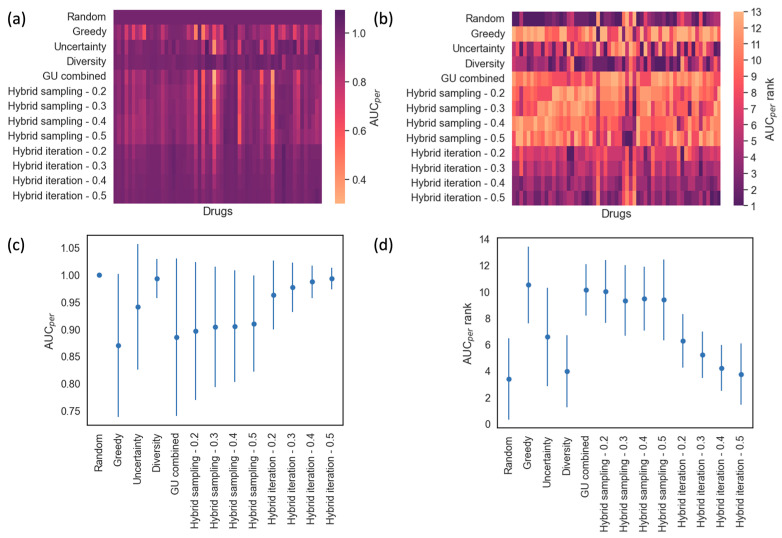
Drug response prediction performance across all methods and drugs. (**a**) Heat map showing the mean AUC_per_ values obtained for all sampling methods and drugs. (**b**) Heat map showing the AUC_per_ rank for each sampling method and drug. The method with the highest AUC_per_ for a particular drug receives the lowest AUC_per_ rank. (**c**) Scatter plot showing the mean and standard deviation of AUC_per_ across all drugs for each method. (**d**) Scatter plot showing the mean and standard deviation of AUC_per_ ranks obtained for each method over all drugs.

The drug response modeling performance measured in terms of AUC_per_ for all methods and drugs is summarized by heatmaps and scatter plots in Figure 6. Because the maximum value of R^2^ obtained over all of the iterations can vary from drug to drug, the AUC_per_ of every method is normalized by the AUC_per_ value of ‘Random’ for each drug. Figure 6a shows the average normalized AUC_per_ for each method and drug. The more purple areas indicate higher AUC_per_ values. To determine which method improves the modeling performance faster, the methods are ranked based on AUC_per_ values for each drug to generate AUC_per_ ranks. The method with the highest AUC_per_ value is ranked 1st and the method with the lowest AUC_per_ value is ranked 13th. Figure 6b is a heatmap showing the AUC_per_ ranks assigned for each method over all drugs. The more purple areas indicate methods with lower AUC_per_ ranks, hence higher AUC_per_. Figure 6c,d show the means and standard deviations over all of the drugs for AUC_per_ values and ranks, respectively. These plots help to understand if a particular method is generally better than the other in improving model prediction performance. ‘Random’, ‘Diversity’, ‘Uncertainty’ and ‘Hybrid iteration’ methods show more purple regions than other methods in Figure 6a,b. This is in agreement with Figure 6d, where those methods have better (or lower) ranks than other methods.

To determine if any of the methods performed better than a baseline method, either ‘Greedy’ or ‘Random’, in terms of model performance improvement, two-tailed *t*-tests were conducted between the AUC_per_ value of each method and that of the baseline approach across all drugs. The results of the t-tests are shown in Table 4. If the difference in mean AUC_per_ is positive, the method in consideration (indicated in the first column) performs better and if the difference is negative, the baseline method (i.e., ‘Greedy’ or ‘Random’) performs better. All of the entries in Table 4 with *p*-value < 0.05 and positive mean AUC_per_ differences are indicated in bold. A pairwise Wilcoxon signed-rank test was also conducted between the AUC_per_ rank of each method and that of ‘Greedy’ or ‘Random’. The *p*-values and the differences in the mean ranks are shown in Table 5. The difference in mean ranks is computed in a way that a positive difference indicates the method in consideration performs better than either ‘Random’ or ‘Greedy’. All of the methods with *p*-values < 0.05 and positive average AUC_per_ rank differences are indicated by bold font in Table 5. 

The results in Table 4 and Table 5 show that none of the methods show a statistically significant *p*-value with a positive difference value when compared to ‘Random’, which means that random sampling improves response modeling performance fastest. In addition to random sampling, ‘Diversity’ and ‘Hybrid iteration—0.5’ both outperform all other sampling methods. We can also see that several methods produce statistically significant *p*-values (<0.05) with positive difference values when compared to ‘Greedy’. Specifically, ‘Uncertainty’, ‘Diversity’, ‘Random’, and all of the ‘Hybrid iteration’ methods outperform ‘Greedy’ with statistically significant *p*-values (<0.05).

## 4. Discussion

This study develops and evaluates thirteen active learning approaches for (1) identifying effective treatments and (2) improving the prediction performance of drug response models. This is of paramount importance as the data for building anti-cancer drug response prediction models are generated through pre-clinical drug screening studies and clinical treatment design. This study investigates drug-specific response prediction models for cancer cell lines under several active learning scenarios such as different acquisition functions and hybrid sampling approaches. The rate of identifying hits indicates how fast the algorithm can recognize potential treatment strategies and thereby save considerable time and resources. The rate of improvement in model performance indicates how quickly the algorithm can select suitable samples to effectively train machine learning models to produce reliable drug response prediction models.

Several methods for uncertainty estimation have been used in active learning workflows such as entropy [43,47,49], empirical standard deviation with bootstrapping [53], Bayesian uncertainty estimation [54], least confidence [52], margin sampling [55], and mutual information [43]. In this work, the uncertainty is estimated by computing the standard deviation of prediction values generated from the ensemble of models. This is a straightforward method where the sole purpose of using ensemble models is to estimate prediction uncertainty. Ensemble learning has been used commonly for improving the prediction accuracy by combining prediction results generated by models trained using different data partitions [3] and/or feature subsets/modalities [56,57]. The results generated from the multiple models within the ensemble are fused [58,59,60] using voting mechanisms, such as simply taking the average, to produce final prediction outcomes [58,59,61]. In this work, the uncertainty and mean prediction values estimated from the ensemble models help identify candidate cancer cell lines to obtain response measurements. 

Compared with existing works of active learning for drug response prediction [30,33], our work has made its unique contributions on exploring active learning for different applications. In [33], active learning methods have been developed and evaluated on screening data generated by assays of protein-drug activities. In our work, we investigate active learning for anti-cancer drug response modeling, where drug responses on cancer cell lines are usually measured using viability assays. Compared with [30], which builds response prediction models specific to a particular cell line, we investigate active learning for building drug-specific response models. Cell line-specific models use drug features to make response predictions for new drugs not included in the training set, which makes them useful for developing new drugs. Drug-specific models use cancer features to make response predictions for new cancer cases not included in the training set, which makes them useful for precision oncology applications. Furthermore, [30] mainly evaluates the capability of active learning schemes in identifying responsive treatments, while the model prediction performance has not been thoroughly studied and compared with baselines. On the contrary, we have rigorously evaluated and compared various active learning strategies for both identifying responsive treatments and improving the response prediction performance.

All of the analyses in this study can identify hits much earlier than the ‘Random’ analysis. This means that in a real-world pre-clinical drug screening study, there is a high probability of choosing an effective treatment when using active learning strategies to select candidate samples in comparison to random selection. Additionally, ‘GU combined’ and ‘Hybrid Sampling—0.2’ approaches are also capable of identifying more hits in comparison to a pure ‘Greedy’ approach. The ability to identify hits is more evident in analyses with acquisition functions with ‘Greedy’, ‘Uncertainty’, or a combination of both. For example, ‘GU combined’ performed the best followed by ‘Greedy’, ‘Uncertainty’, ‘Hybrid sampling’ methods, and ‘Hybrid iteration’ methods. ‘Random’ and ‘Diversity’ performed the worst with higher (or worse) AUC_hit_ ranks, as those acquisition functions are not dependent on the candidate set predictions. This could be because when using the ‘Greedy’ acquisition function, models are selectively trained on samples with higher hits and therefore the models are able to identify candidate set samples with higher hits in subsequent iterations. Adding an uncertainty term to the ‘Greedy’ acquisition function produces the ‘GU combined’ acquisition function. Since ‘Uncertainty’ also uses candidate set predictions, a combination of both of the methods further helps in identifying hits. The lower the influence of the ‘Random’ acquisition function, the better the performance in hit identification, as can be seen in both the hybrid approaches.

On the other hand, all of the analyses not containing or with a lower contribution of the ‘Greedy’ acquisition function showed better model performance with lower (or better) AUC_per_ ranks. For example, ‘Random’ performed the best, followed by ’Diversity’, ‘Uncertainty’, all ‘Hybrid iteration’ methods, and some ‘Hybrid sampling’ methods. This means that since the ‘Greedy’ acquisition function selectively chooses a candidate set sample with higher hits, the model will not encounter samples with lower hits during training and therefore the overall model performance is lower for those acquisition functions with a higher contribution of ‘Greedy’. This is evident in both the hybrid approaches where a lower contribution of ‘Greedy’ shows a better model performance improvement. It is interesting to note that the performance of ‘Uncertainty’ is almost the same with both AUC_hit_ and AUC_per_ around five to seven. This means that ‘Uncertainty’ contributes almost the same in identifying hits as well as in improving the model performance. 

In an active learning iteration, the next batch of cell lines to be experimented with will be selected using a sampling strategy, for which the best option depends on the objective of conducting active learning. If the goal is to find effective treatments, which are cancer cell lines responding to a drug treatment, our analysis results indicate ‘GU combined’ can be the top choice for a sampling method. It helped to identify responsive treatments more quickly in the study, as shown in Figure 5, Table 2 and Table 3. On the other hand, if the goal is to improve the prediction accuracy for the drug response, ‘Random’ and ‘Diversity’ can be the top choices for the sampling method, based on our analysis results shown in Figure 6 and Table 4 and Table 5. These methods select samples that improve the model prediction performance more quickly.

Active learning strategies may also be extended to cancer patient data [60,62,63], possibly with the assistance of transfer learning [3]. Via transfer learning, a model pretrained on cell line drug response data can be used in the active learning procedure with patient data. In every iteration of the active learning procedure, the pretrained cell line response model will be refined using available patient response data to make predictions for candidate patients. Then, the next batch of patients to be treated by the drug can be selected by considering the prediction results. The overall active learning workflow on patient data will be similar to Figure 1. Active learning can also be applied solely on patient data without transfer learning using pretrained cell line response models, in which every iteration will train response models from scratch based on patient data only. But transfer learning is expected to improve the predictions on patients as it leverages the relatively abundant drug response information in cell line data. The Cancer Genome Atlas (TCGA) contains primary patient tumors with molecular profiles and clinical drug response data [64], which can be used to test the active learning workflows for patient tumors. There is also the potential for applying active learning in patient selection in clinical trials/practice, where patients need to start treatments as soon as possible, even before tumor molecular profiles are available. To achieve that, response prediction models need to be built based on cancer data/features other than molecular profiles, such as radiology images, pathology images, and clinical records.

## 5. Conclusions

This study, investigating thirteen active learning analyses conducted on 57 drugs for identifying effective treatment strategies and for improving machine learning model prediction performance, has made several unique contributions. The work performs a comprehensive investigation on multiple active learning techniques for anti-cancer drug response prediction on drug-specific models where the response of various cell lines is predicted for specific drug treatments. Several sampling techniques have been investigated based on different acquisition functions such as ‘Greedy’, ‘Uncertainty’, ‘Diversity’, ‘GU combined’, and ‘Random’. In addition, several hybrid approaches have been devised to further explore their advantages in identifying potential candidate experiments as well as for improving the model performance. Finally, the performance of active learning workflows utilizing these sampling techniques were evaluated using a set of novel experimental procedures and performance metrics. We have demonstrated that all of the active learning strategies are more effective in identifying hits than random sampling. On the other hand, random sampling and active learning strategies using diversity and uncertainty acquisition functions improve model performance compared to other active learning strategies.

## Figures and Tables

**Table 2:** Results of *t*-tests conducted on AUC_hit_ values between each method and a ‘Greedy’ or ‘Random’ baseline approach.

Analysis ‘X’	*p*-Value with ‘Greedy’	(AUC_hit_)_X_ − (AUC_hit_)_Greedy_	*p*-Value with ‘Random’	(AUC_hit_)_X_ − (AUC_hit_)_Random_
Greedy	-	-	**1.02** ** × 10^−39^**	**0.24**
Random	1.02 × 10^−39^	−0.24	-	-
Uncertainty	0.55	0.01	**9.13** ** × 10^−24^**	**0.26**
GU combined	**0.015**	**0.04**	**1.22** ** × 10^−40^**	**0.29**
Diversity	1.19 × 10^−25^	−0.19	**8.54** ** × 10^−8^**	**0.05**
Hybrid sampling—0.2	0.15	0.03	**6.44** ** × 10^−38^**	**0.27**
Hybrid sampling—0.3	0.43	0.01	**2.85** ** × 10^−36^**	**0.26**
Hybrid sampling—0.4	0.99	2.0 × 10^−4^	**1.71** ** × 10^−34^**	**0.24**
Hybrid sampling—0.5	0.33	−0.02	**1.89** ** × 10^−32^**	**0.23**
Hybrid iteration—0.2	1.00 × 10^−4^	−0.06	**9.95** ** × 10^−42^**	**0.18**
Hybrid iteration—0.3	1.00 × 10^−10^	−0.10	**1.51** ** × 10^−40^**	**0.15**
Hybrid iteration—0.4	1.06 × 10^−18^	−0.13	**4.17** ** × 10^−41^**	**0.11**
Hybrid iteration—0.5	8.16 × 10^−26^	−0.16	**1.82** ** × 10^−40^**	**0.07**

**Table 3:** Results of Wilcoxon signed-rank tests conducted between each method and a ‘Greedy’ or ‘Random’ based on AUC_hit_ rank values.

Analysis ‘X’	*p*-Value with ‘Greedy’	(AUC_hit_ Rank)_Greedy_ − (AUC_hit_ Rank)_X_	*p*-Value with ‘Random’	(AUC_hit_ rank)_Random_ − (AUC_hit_ Rank)_X_
Greedy	-	-	**4.36 × 10^−11^**	**8.79**
Random	4.36 × 10^−11^	−8.79	-	-
Uncertainty	0.35	−0.72	**4.97** ** × 10^−11^**	**8.07**
GU combined	**8.30** ** × 10^−9^**	**2.45**	**1.91** ** × 10^−11^**	**11.24**
Diversity	4.47 × 10^−11^	−7.79	**6.16** ** × 10^−8^**	**1.00**
Hybrid sampling—0.2	**7.00** ** × 10^−3^**	**1.12**	**3.85** ** × 10^−12^**	**9.91**
Hybrid sampling—0.3	0.79	−0.09	**5.31** ** × 10^−12^**	**8.70**
Hybrid sampling—0.4	5.00 × 10^−4^	−1.17	**7.12** ** × 10^−12^**	**7.61**
Hybrid sampling—0.5	4.26 × 10^−7^	−2.52	**1.20** ** × 10^−11^**	**6.26**
Hybrid iteration—0.2	1.10 × 10^−9^	−3.35	**1.66** ** × 10^−11^**	**5.44**
Hybrid iteration—0.3	1.00 × 10^−10^	−4.77	**4.64** ** × 10^−12^**	**4.02**
Hybrid iteration—0.4	4.80 × 10^−11^	−5.89	**5.45** ** × 10^−12^**	**2.89**
Hybrid iteration—0.5	4.52 × 10^−11^	−7.02	**6.06** ** × 10^−12^**	**1.77**

**Table 4:** Results of t-tests conducted between AUC_per_ values of active learning methods and those from ‘Greedy’ or ‘Random’.

Analysis ‘X’	*p*-Value with ‘Greedy’	(AUC_per_)_X_ − (AUC_per_)_Greedy_	*p*-Value with ‘Random’	(AUC_per_)_X_ − (AUC_per_)_Random_
Greedy	-	-	2.90 × 10^−11^	−0.13
Random	**2.90** ** ×** **10^−11^**	**0.13**	-	-
Uncertainty	**3.00** ** ×** **10^−3^**	**0.07**	2.5 × 10^−4^	−0.06
GU combined	0.57	0.02	3.61 × 10^−8^	−0.11
Diversity	**6.00** ** ×** **10^−10^**	**0.12**	0.19	−6.40 × 10^−3^
Hybrid sampling—0.2	0.28	0.03	1.54 × 10^−8^	−0.10
Hybrid sampling—0.3	0.14	0.03	2.50 × 10^−9^	−0.09
Hybrid sampling—0.4	0.12	0.04	3.00 × 10^−10^	−0.09
Hybrid sampling—0.5	0.06	0.04	1.03 × 10^−11^	−0.09
Hybrid iteration—0.2	**5.50** ** ×** **10^−6^**	**0.09**	3.30 × 10^−5^	−0.04
Hybrid iteration—0.3	**7.81** ** ×** **10^−8^**	**0.11**	2.50 × 10^−4^	−0.02
Hybrid iteration—0.4	**2.40** ** ×** **10^−9^**	**0.11**	2.10 × 10^−3^	−0.01
Hybrid iteration—0.5	**3.00** ** ×** **10^−10^**	**0.12**	0.01	−0.006

**Table 5:** Results from the Wilcoxon signed-rank tests conducted between AUC_per_ ranks of active learning methods and those of ‘Greedy’ or ‘Random’.

**Analysis ‘X’**	***p*** **-Value with ‘Greedy’**	**(AUC_per_ Rank)_Greedy_ − ** **(AUC_per_ Rank)_X_**	***p*** **-Value with ‘Random’**	**(AUC_per_ Rank)_Random_ − ** **(AUC_per_ Rank)_X_**
Greedy	-	-	8.60 × 10^−9^	−7.12
Random	**8.60** ** ×** **10^−9^**	**7.12**	-	-
Uncertainty	**2.00** ** ×** **10^−5^**	**3.94**	6.30 × 10^−6^	−3.17
GU combined	0.07	0.38	9.00 × 10^−10^	−6.73
Diversity	**2.00** ** ×** **10^−10^**	**6.54**	0.03	−0.57
Hybrid sampling—0.2	0.54	0.50	1.44 × 10^−8^	−6.61
Hybrid sampling—0.3	**0.02**	**1.19**	1.18 × 10^−7^	−5.93
Hybrid sampling—0.4	**0.03**	**1.05**	2.50 × 10^−8^	−6.07
Hybrid sampling—0.5	**0.04**	**1.14**	1.72 × 10^−7^	−5.98
Hybrid iteration—0.2	**3.90** ** ×** **10^−9^**	**4.24**	3.2 × 10^−5^	−2.87
Hybrid iteration—0.3	**4.00** ** ×** **10^−9^**	**5.29**	5.00 × 10^−4^	−1.82
Hybrid iteration—0.4	**2.60** ** ×** **10^−9^**	**6.29**	0.02	−0.82
Hybrid iteration—0.5	**5.40** ** ×** **10^−9^**	**6.77**	0.15	−0.35

## Data Availability

The data used for this work is from the public Cancer Therapeutics Response Portal available at https://portals.broadinstitute.org/ctrp.v2.1/ (accessed February 2018), and from the Cancer Cell Line Encyclopedia available at https://depmap.org/portal/ccle/ (accessed February 2018). The models were trained using the Python package LightGBM, version 3.2.1 available here: https://lightgbm.readthedocs.io/en/latest/index.html (accessed January 2023).

## Supplementary Materials

The following supporting information can be downloaded at: https://www.mdpi.com/article/10.3390/cancers16030530/s1, Table S1: The list of drugs used in the active learning workflows along with their Mechanisms of Action.
