# Supplementary material for: A Comprehensive Investigation of Active Learning Strategies for Conducting Anti-Cancer Drug Screening
Source: Cancers (Basel). 2024 Jan 26;16(3):530. doi: 10.3390/cancers16030530 (PMC10854925; doi:10.3390/cancers16030530)
Supplement: Supplementary file 1 [file cancers-16-00530-s001.zip › cancers-2771580-supplementary.pdf]

Table S1: The list of drugs used in the active learning workflows along with their Mechanisms of Action

	Drug name	Mechanism of Action (MoA)
1	Oligomycin-a	ATP SYNTHASE INHIBITOR|ATPASE INHIBITOR
2	Ouabain	ATPASE INHIBITOR
3	Alisertib	AURORA KINASE INHIBITOR
4	Barasertib	AURORA KINASE INHIBITOR
5	ABT-263	BCL INHIBITOR
6	Dasatinib	BCR-ABL KINASE INHIBITOR|EPHRIN INHIBITOR|KIT INHIBITOR|PDGFR TYROSINE KINASE RECEPTOR INHIBITOR|SRC INHIBITOR|TYROSINE KINASE INHIBITOR
7	SNS-032	CDK INHIBITOR|CELL CYCLE INHIBITOR|MCL1 INHIBITOR
8	Rigosertib	CELL CYCLE INHIBITOR|PLK INHIBITOR
9	AZD7762	CHK INHIBITOR
10	Methotrexate	DIHYDROFOLATE REDUCTASE INHIBITOR
11	Mitomycin-C	DNA ALKYLATING AGENT|DNA SYNTHESIS INHIBITOR
12	Decitabine	DNA METHYLTRANSFERASE INHIBITOR
13	BIBW2992	EGFR INHIBITOR
14	Neratinib	EGFR INHIBITOR
15	KPT185	EXPORTIN ANTAGONIST
16	Panobinostat	HDAC INHIBITOR
17	SNX-2112	HSP INHIBITOR
18	17-AAG	HSP INHIBITOR
19	SB-743921	KINESIN-LIKE SPINDLE PROTEIN INHIBITOR
20	RITA	MDM INHIBITOR
21	AZD8055	MTOR INHIBITOR
22	NVP-BEZ235	MTOR INHIBITOR|PI3K INHIBITOR
23	GMX-1778	NAMPT INHIBITOR
24	CAY10618	NAMPT INHIBITOR
25	FK866	NIACINAMIDE PHOSPHORIBOSYLTRANSFERASE INHIBITOR
26	GSK461364	PLK INHIBITOR
27	BI-2536	PLK INHIBITOR
28	MLN2238	PROTEASOME INHIBITOR
29	Brefeldin-A	PROTEIN SYNTHESIS INHIBITOR

30	Omacetaxine-mepesuccinate-	PROTEIN SYNTHESIS INHIBITOR
31	Cytarabine	RIBONUCLEOTIDE REDUCTASE INHIBITOR
32	Gemcitabine	RIBONUCLEOTIDE REDUCTASE INHIBITOR
33	Clofarabine	RIBONUCLEOTIDE REDUCTASE INHIBITOR
34	Triptolide	RNA POLYMERASE INHIBITOR
35	KX2-391	SRC INHIBITOR|TUBULIN POLYMERIZATION INHIBITOR
36	YM-155	SURVIVIN INHIBITOR
37	Doxorubicin	TOPOISOMERASE INHIBITOR
38	SN-38	TOPOISOMERASE INHIBITOR
39	Topotecan	TOPOISOMERASE INHIBITOR
40	Parbendazole	TUBULIN POLYMERIZATION INHIBITOR
41	Vincristine	TUBULIN POLYMERIZATION INHIBITOR
42	Paclitaxel	TUBULIN POLYMERIZATION INHIBITOR
43	Foretinib	VEGFR INHIBITOR
44	BRD-K97651142	
45	CR-1-31B	
46	Cucurbitacin-I	
47	1S-3R-RSL-3	
48	ML162	
49	ML210	
50	Apicidin	
51	Austocystin-d	
52	Narciclasine	
53	Obatoclax-Mesylate	
54	Pluripotin	
55	Rapamycin	
56	SR-II-138A	
57	Triazolothiadiazine	
